# Biodegradation of Ochratoxin A by Bacterial Strains Isolated from Vineyard Soils

**DOI:** 10.3390/toxins7124864

**Published:** 2015-11-27

**Authors:** Palmira De Bellis, Mariana Tristezza, Miriam Haidukowski, Francesca Fanelli, Angelo Sisto, Giuseppina Mulè, Francesco Grieco

**Affiliations:** 1 Institute of Sciences of Food Production, National Research Council, Unit of Bari, via Amendola 122/O, Bari 70126, Italy; mirella.debellis@ispa.cnr.it (P.D.B.); miriam.haidukowski@ispa.cnr.it (M.H.); francesca.fanelli@ispa.cnr.it (F.F.); angelo.sisto@ispa.cnr.it (A.S.); giuseppina.mule@ispa.cnr.it (G.M.); 2 Institute of Sciences of Food Production, National Research Council, Unit of Lecce, via Provinciale Lecce-Monteroni, Lecce 73100, Italy; mariana.tristezza@unifg.it

**Keywords:** ochratoxin, biodegradation, soil bacteria, detoxification, *Acinetobacter*

## Abstract

Ochratoxin A (OTA) is a mycotoxin with a main nephrotoxic activity contaminating several foodstuffs. In the present report, five soil samples collected from OTA-contaminated vineyards were screened to isolate microorganisms able to biodegrade OTA. When cultivated in OTA-supplemented medium, OTA was converted in OTα by 225 bacterial isolates. To reveal clonal relationships between isolates, molecular typing by using an automated rep-PCR system was carried out, thus showing the presence of 27 different strains (rep-PCR profiles). The 16S-rRNA gene sequence analysis of an isolate representative of each rep-PCR profiles indicated that they belonged to five bacterial genera, namely *Pseudomonas*, *Leclercia*, *Pantoea*, *Enterobacter*, and *Acinetobacter*. However, further evaluation of OTA-degrading activity by the 27 strains revealed that only *Acinetobacter calcoaceticus* strain 396.1 and *Acinetobacter* sp. strain neg1, consistently conserved the above property; their further characterization showed that they were able to convert 82% and 91% OTA into OTα in six days at 24 °C, respectively. The presence of OTα, as the unique OTA-degradation product was confirmed by LC-HRMS. This is the first report on OTA biodegradation by bacterial strains isolated from agricultural soils and carried out under aerobic conditions and moderate temperatures. These microorganisms might be used to detoxify OTA-contaminated feed and could be a new source of gene(s) for the development of a novel enzymatic detoxification system.

## 1. Introduction

Mycotoxins are natural contaminants in foodstuffs that, according to the Food and Agricultural Organization (FAO), contaminate more than 25% of the world’s commodities [1]. Among more than 300 already known mycotoxins [2,3], ochratoxin A (OTA) is one of the most important not only for its toxicological potential but also because of its extensive distribution. OTA is nephrotoxic, teratogenic, embryotoxic, immunotoxic, genotoxic, and neurotoxic in animals and humans [4,5]. Moreover, OTA is considered a possible human carcinogen and is classified in Group 2B by the International Agency of Research on Cancer [6]. OTA is produced by species belonging to *Aspergillus* and *Penicillium* genera, mainly *Aspergillus niger*, *A. ochraceus*, *A. carbonarius*, and *Penicillium verrucosum*. Ochratoxin A, an isocoumarin derivative linked by its carboxyl group to l-β-phenylalanine, is one of the most relevant mycotoxins because of both its toxicological potential and widespread distribution [7]. Its occurrence has been reported in a wide variety of food and beverages including cereals, cocoa, olives, coffee, beer, and spices [4,8,9,10]. Grapes and their derived products, such as grape juice, dried vine fruits, and wine, are frequently contaminated with this toxin [11,12] and are among the major sources of OTA dietary exposure [13]. OTA contamination severity in vineyards depends on several factors, including environmental conditions, vintage, vineyard location, cropping system [14,15,16,17], soil type, characteristics of the grapes [18], and competitive growth of microorganisms [19]. For example, OTA occurrence in Europe is higher in wines from southern regions because of the warmer climates [11] and red wines are contaminated more frequently than white ones probably due to the different winemaking methods [20,21].

Due to the impact of this mycotoxin on health, preventive measures for the reduction of fungal growth and mycotoxin levels have received much attention in recent years. The usual methods to reduce the levels of OTA comprised several approaches, such as physical treatment, addition with absorbent materials, or solvent extraction. Nevertheless, these systems are often expensive and they cause organoleptic changes in treated foodstuffs. Decontamination or detoxification procedures have been considered and, in particular, several physical, chemical, or biological approaches have been proposed for OTA reduction in musts and wine [21]. Biodegradation is considered one of the most promising approaches to control mycotoxins. Several enzymes may be involved in the microbiological degradation of OTA [22]. However, little information is available and very few of them have been purified and characterized. The first reported protease able to hydrolyze OTA was carboxypeptidase A (CPA) (EC 3.4.17.1) from bovine pancreas, thus releasing l-β-phenylalanine and ochratoxin α (OTα) [23,24,25]. OTα is considered a non-toxic compound and has an elimination half-life 10-times shorter than OTA [26]. Several studies have reported degradation of ochratoxin A by different microorganisms, such as *Phenylobacterium immobile* [27], *Acinetobacter calcoaceticus* [28], *Trichosporon mycotoxinivorans* [29], *Brevibacterium* spp. [30], *Pediococcus parvulus* [31], and *Aspergillus* spp. [32,33,34]. Several reports also showed that agricultural soils could represent an interesting source of microorganisms with ability to degrade mycotoxins [35,36]. In fact, soil bacteria, such as *Pseudomonas* spp. and *Acinetobacter* spp. are capable of converting a large number of aromatic compounds, thus playing an important role in the biodegradation of toxic molecules in contaminated soils [37]. For example, Shima and coworkers [38] identified from a soil sample a Gram-negative bacteria able to aerobically metabolize the mycotoxin deoxynivalenol (DON) into 3-keto-4-deoxynivalenol. Additionally, bacterial strains of soil origin belonging to *Nocardioides* and *Devosia* genera were characterized for their capacity to degrade DON to 3-epi-deoxynivalenol [36], whereas Islam *et al.* [35] isolated from agricultural soils bacterial populations able to biodegrade DON under aerobic conditions and moderate temperatures via a de-epoxydation mechanism. With this regard, in our knowledge, this is the first study aimed to evaluate degradation of ochratoxin A in soil samples from vineyards contaminated by high levels of OTA in order to isolate new microorganisms with OTA-degrading ability.

## 2. Results

### 2.1. Degradation Activity of Soil and Branch Samples

**Figure 1:**
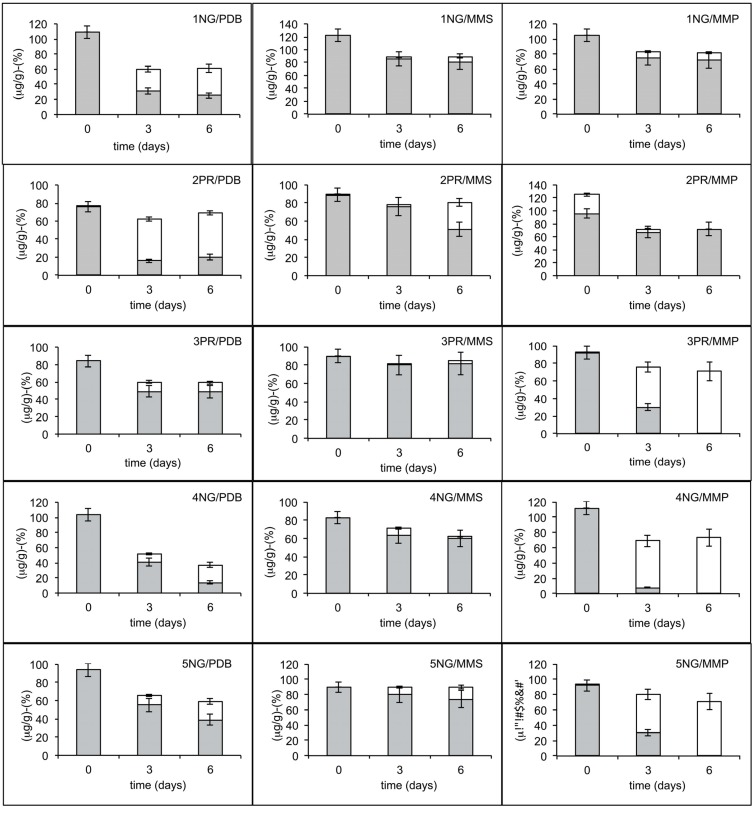
Degradation of OTA (**grey**) in OTα (**white**) by microbial populations associated to five soil samples in three liquid substrates.

**Figure 2:**
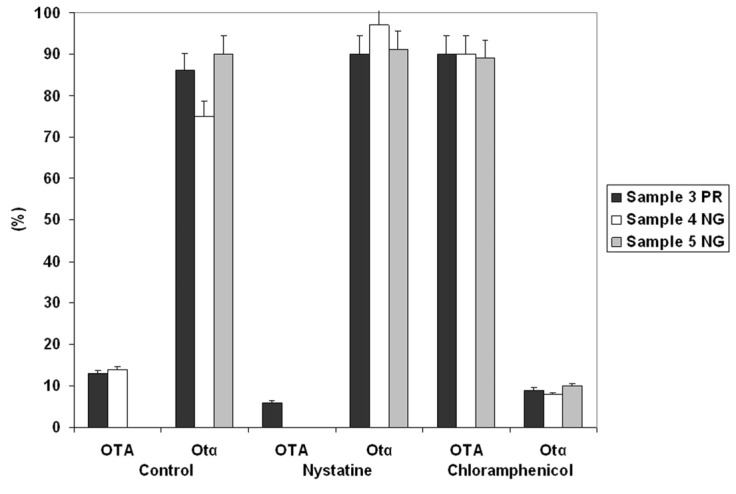
Ochratoxin A degradation by microbial populations associated with the three soil samples respectively cultured in presence of nystatine or chloramphenicol. The OTA degradation in absence of antibiotic addition is also shown.

Soil and branch samples were first analyzed in three different culture media to promote the growth of fungi, yeast, and bacteria in the presence of OTA. The results shown in Figure 1 indicate that microbial populations from three soil samples, when grown in Minimal Medium Peptone (MMP) substrate, achieved the total degradation of OTA to OTα in six days, while cultures growing in Potatto Dextrose Broth (PDB) and Minimal Medium Sucrose MMS substrates did not attain a complete biodegradation of OTA. The cultures also failed the biodegradation of OTA in MM (data not shown). The microbial populations associated to branches did not show any ability to biodegrade the OTA (data not shown). An aliquot of each one of the three soil suspensions with OTA-degrading ability was then used to inoculate MMP and PDB supplemented with 10 µg/mL of OTA and a given antibiotic or an antifungal agent. The results (Figure 2) revealed the bacterial nature of microorganisms with degrading properties present within each microbial soil population, since this degrading activity was not modified by the presence of nystatine, but was inhibited by the addition of chloramphenicol.

**Figure 3:**
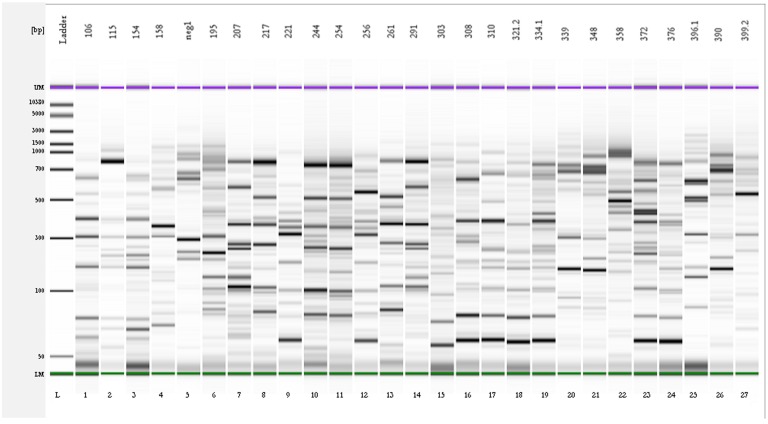
Gel-like image obtained using the Agilent Expert software of the 27 different rep-PCR profiles identified after the analysis of the 225 OTA-degrading isolates. The profiles were obtained using primers REP-1R-Dt/REP-2R-Dt. L: DNA 7500 ladder.

### 2.2. Genotypic Characterization and Identification of Bacteria from OTA-Degrading Soils

The results obtained by chemical analysis showed that bacterial populations from soil samples 3PR, 4NG, and 5NG included isolates able to degrade OTA to OTα, while this property was not detected in populations associated with soil samples 1NG and 2PR. On the basis of this evidence, a total of 225 bacterial isolates with degrading activity were obtained from those three soil samples and then investigated for possible clonal relationships, using the rep-PCR technique. Twenty-seven different rep-PCR patterns, each associated with a number of isolates ranging from 1 to 64, were obtained using the REP primers (Figure 3). In particular, 13 profiles differentiated the isolates from soil sample 3PR, while six and eight patterns distinguished those from soil samples 4NG and 5NG, respectively.

An isolate representative of each rep-PCR profile was identified by sequencing about 1400 bp of the 16S rRNA gene. Primer pair P0/P6 was suitable for the amplification of the 16S rRNA gene from isolates belonging to the genus *Acinetobacter*, while the primer pair 27F-YM/1492r provided better results for the other isolates. Database searches with these sequences showed identities >99% with type strain sequences available in database, leading to the assignment of the following bacterial species: *Acinetobacter calcoaceticus*, *Pseudomonas taiwanensis*, *Pseudomonas reinekei*, *Pseudomonas koreensis*, *Leclercia adecarboxylata*, *Pantoea agglomenans*, *Enterobacter aerogenes*, and *Enterobacter xiangfangensis* (Table 1). The genus *Pseudomonas* was the one more frequently isolated from each soil and *Pseudomonas koreensis* was the only detected species from two soils (3PR and 5NG). *Acinetobacter* sp. strain neg1 was an exception because its 16S rRNA gene showed the best alignment with *Acinetobacter baumanni* but at a percentage of identity <99% (*i.e.*, 98.29% and four gaps); in fact, this strain was assigned to a new species on the basis of genomic data (Genome GenBank accession number JSZD00000000; [39]). The 16S rRNA gene sequence of strain 396.1, due to its relevance as an OTA-degrading strain, was deposited under GenBank accession number KR856228.

**Table 1:** Bacterial strains isolated from OTA-degrading soils.

Species	Strain (REP-PCR Profile)	Isolation Soil
*Pseudomonas taiwanensis*	106 (1), 115 (2), 154 (3)	4NG
*Leclercia adecarboxylata*	158 (4)	4NG
*Acinetobacter* sp.	neg1 (5)	4NG
*Pantoea agglomerans*	195 (6)	4NG
*Pseudomonas reinekei*	207 (7), 217 (8), 261 (13), 291 (14)	5NG	
*Pseudomonas koreensis*	221 (9), 244 (10), 254 (11), 256 (12)	5NG	
	303 (15), 308 (16), 310 (17), 321.2 (18), 334.1 (19), 372 (23)	3PR	
*Enterobacter aerogenes*	339 (20), 348 (21), 358 (22), 376 (24), 390 (26)	3PR
*Acinetobacter calcoaceticus*	396.1 (25)	3PR
*Enterobacter xiangfangensis*	399.2 (27)	3PR

### 2.3. OTA Degradation by Acinetobacter spp.

**Figure 4:**
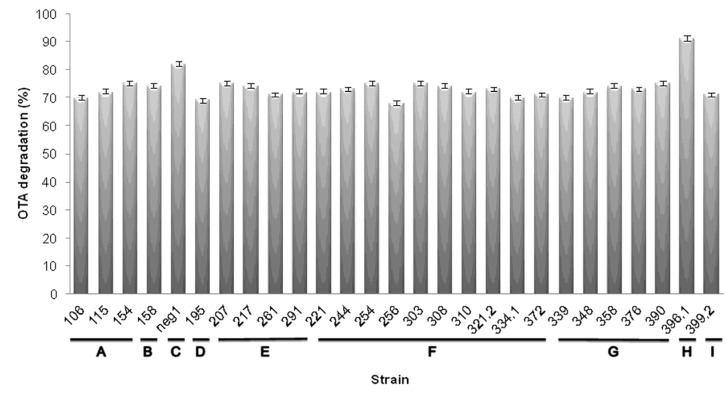
Percentage of OTA degradation by the twenty-seven strains identified by the REP-PCR analysis. Cultures were incubated at 24 °C and mycotoxin degradation was evaluated by HPLC-FLD assay after six days. Bars represent standard errors. (**A**) *Pseudomonas taiwanensis*; (**B**) *Leclercia adecarboxylata*; (**C**) *Acinetobacter* sp. neg1; (**D**) *Pantoea agglomerans*; (**E**) *Pseudomonas reinekei*; (**F**) *Pseudomonas koreensis*; (**G**) *Enterobacter aerogenes*; (**H**) *Acinetobacter calcoaceticus*; and (**I**) *Enterobacter xiangfangensis*.

**Figure 5:**
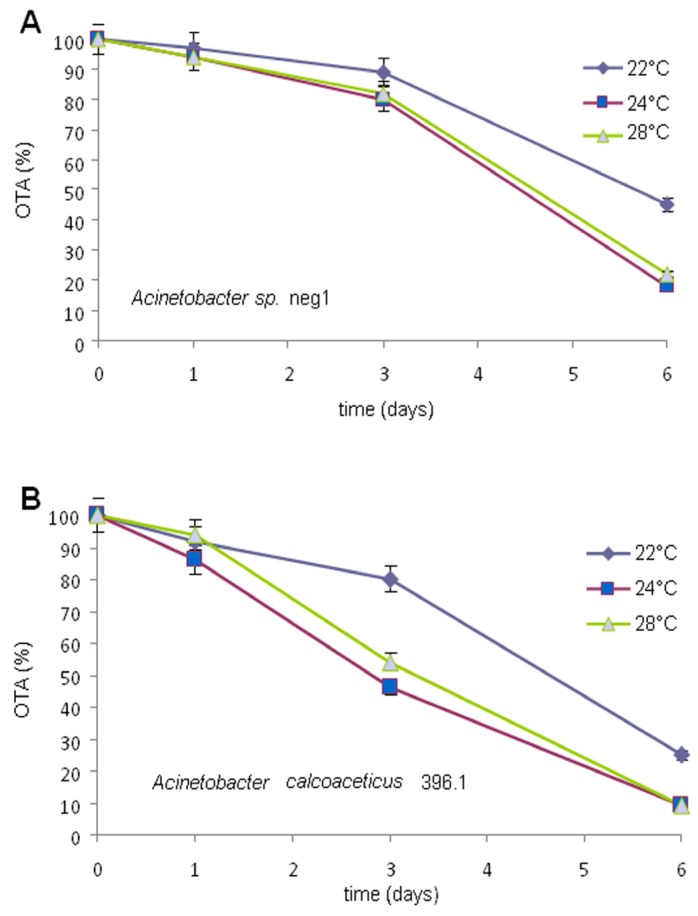
OTA degradation by *Acinetobacter* sp. neg1 (**A**) and *A. calcoaceticus* 396.1; and (**B**) in MMP medium added with 1 µg/mL of OTA. Cultures were incubated at 22, 24 and 28 °C and mycotoxin degradation was evaluated by HPLC-FLD assay at 13 and six days post inoculation.

**Figure 6:**
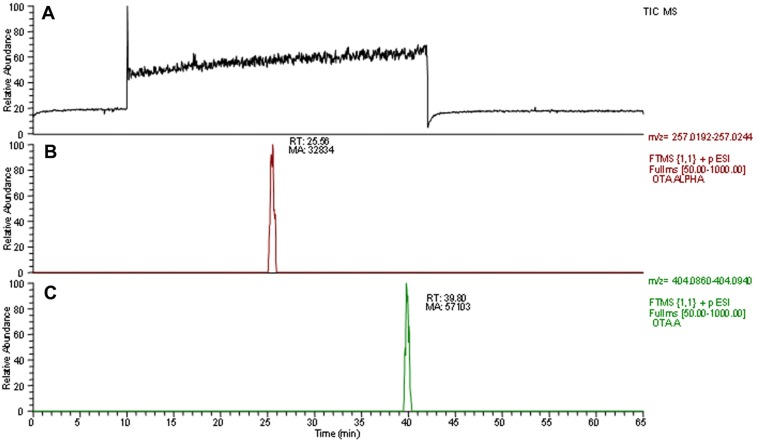
LC-HR-MS Total Ion Current (TIC) and extracted ion chromatograms (XIC) filtered on the accurate mass of ochratoxin A and ochratoxin alpha from the supernatant of liquid culture sample added with ochratoxin A and inoculated *Acinetobacter* sp. neg1. In the upper panels are reported (**A**) the full mass chromatogram; (**B**) the extracted ion chromatogram filtered on the accurate mass of ochratoxin alpha; and (**C**) the extracted ion chromatogram filtered on the accurate mass of ochratoxin A.

The twenty-seven identified strains were preliminarily characterized for their ability in OTA degradation. A marked degradation of OTA level was observed after six days (Figure 4). However, when these strains were further characterized for their ability in OTA conversion, the only strains that maintained the OTA-degrading ability were the neg1 and 396.1, both belonging to the *Acinetobacter* genus. These two strains were from two out of three soil samples showing degrading activity, in particular, *Acinetobacter* sp. strain neg1 from soil 4NG and *A. calcoaceticus* strain 396.1 from 3PR. A significant degradation of OTA level was observed over six days. The results of the analyses revealed that these strains (viable cells) degraded OTA to OTα. The average degradation levels of OTA with respect to the control (with 100% OTA) were 20% (RSD, 20%) after three days and 82% (RSD, 12%) after six days for *Acinetobacter* sp. strain neg1, while for *A. calcoaceticus* strain 396.1 were 54% (RSD, 12%) after three days and 91% (RSD, 1%) after six days at 24 °C (Figure 5). Based on our evidence, when tested in a temperature range spanning from 22 to 28 °C, the bacterial degrading activity was slightly more efficient at 24 °C. Degrading activity was even functional after one day of incubation, when, for example at 24 °C, 6% and 14% of OTA resulted in being degraded by strain neg1 and strain 396.1, respectively. Results confirmed the ability of enzymes produced by strains neg1 and 396.1 of degrading OTA to OTα. According to the molecular structure of OTA, the microbial degradation through the hydrolysis of the amide bond results in l-β-phenylalanine molecule and OTα [23,24,25]. The production of OTα metabolite was ascertained by HPLC-HRMS (Figure 6). Our analyses did not reveal the presence of l-β-phenylalanine, possibly indicating that l-β-phenylalanine was immediately used or further degraded by bacterial cells.

## 3. Discussion

To date, no biological treatment is currently employed to reduce the content of OTA in foods, beverages, and feed. To fulfill this urgent demand and because of previous evidence showing [30,40] that soil bacteria are capable of converting a wide assortment of aromatic compounds, we decided to analyze the fate of OTA in the presence of bacterial populations associated with soil samples. Since it has been shown that OTA can absorb relatively strongly to soil organic matter [41], the soil samples used in the present study were collected from vineyards infected by ochratoxigenic fungi and contaminated by high levels of OTA [42]. A rapid degradation of OTA in soil was demonstrated by Mortensen and coworkers [43], who showed that the degradation was faster in planted soil compared to unplanted soil, possibly due to a higher microbial activity in the rhizosphere of the planted soil [44]. In fact, our analyses confirmed an OTA-degrading activity also in our collected soil samples. In our initial screening steps, upwards of 144 h of incubation were required to achieve >70% OTA degradation by bacterial populations associated to the analyzed soil samples. The extent of the time required for toxin conversion can be explained by the presence of quickly growing microbes in artificial media that provoke a suppressive action on the growth of the OTA degrading bacterial strains [35,45]. The use of antibiotics and antifungal compounds with different modes of actions [46] allowed us to determine the bacterial nature of the OTA-degrading microbial populations associated to the soil samples. In this regard, the present investigation also describes the selective isolation from media supplemented with OTA of 225 soil bacterial isolates that, in a first screening test, showed OTA-degrading activity. A rep-PCR analysis enabled us to reveal the clonal relationships among the isolates leading to the detection of 27 different rep-PCR profiles, each associated to a variable number of isolates. The identification at the species level of 27 representative isolates, each characterized by a different rep-PCR profile, indicated that the 225 isolates belonged to nine different bacterial species. Three of these were of the *Pseudomonas* genus, while the other belonged to the genera *Acinetobacter*, *Enterobacter*, *Pantoea*, and *Leclercia*. Therefore, these results could suggest the presence in the analyzed soils of a relevant biodiversity, at the species and strain levels, of bacteria able to grow in the presence of OTA and to degrade this toxic compound. However, when the 27 representative isolates were further analyzed to confirm their OTA-degrading activity only two of them, namely *Acinetobacter calcoaceticus* (strain 396.1) and *Acinetobacter* sp. (strain neg1; [39]), consistently conserved this capability which, on the contrary, was definitely lost by the other strains.

The two abovementioned bacterial strains isolated from agricultural soil have confirmed their own capacity to convert OTA to OTα in aerobic conditions and at moderate temperatures. The loss of OTA-degrading activity by the other strains could be possibly due to the loss of genes responsible for this capability occurring during subculturing under not selective conditions in media without OTA. A similar behavior has been already described for OTA degradation by *Lactobacillus vitulinus* [29], chlorinated biphenyls conversion by *Acinetobacter* sp. strain B6 [47], and methyl parathion removal by *Pseudomonas* sp. [48]. Therefore, the two *Acinetobacter* strains, *i.e.*, *A. calcoaceticus* (strain 396.1) and *Acinetobacter* sp. (strain neg1), were selected for further investigations.

OTA-degrading activity of *A. calcoaceticus* was also described in a previous investigation in which strain NRRL B-551 was shown to be able to degrade OTA in ethanol minimal salts medium with an initial concentration of 10 μg/mL at both 25 and 30 °C [28]; nonetheless, it is important to note that the OTA-degrading strain neg1 isolated in this study, based on genomic data [39], belongs to a new species.

A relevant interest should be addressed to the abovementioned *Acinetobacter* strains, isolated in this study, because of the high percentage of OTA removal. In addition, it should be considered the ecological importance of the genus *Acinetobacter* recently used as a model microorganism for studies in environmental microbiology and industrial production of chemicals [49]. Furthermore, the OTA degradation mechanism of both *Acinetobacter* strains was also denoted by a technologically relevant feature, that is, the ability to efficiently convert OTA in OTα at a moderate temperature (<25 °C). Indeed, with the exception of that concerning *Phenylobacterium immobile* [27], the available reports about OTA degradation by bacterial strains indicated that *Bacillus licheniformis* [50], lactic acid bacteria [51,52]; *Brevibacterium* spp. [30], *Bacillus amyloliquefaciens* [53], and *Pediococcus parvulus* [31] were able to convert OTA at a temperature equal or higher than 30 °C. The advantage of the present finding is that OTA conversion activity of the culture was optimal in a temperature range that matches the temperature range of agronomical crops. Therefore, it is reasonable to hypothesize that the gene(s)/enzyme(s) responsible for the activity have potential application for enzymatic detoxification of OTA in food and feed of vegetal origin [22].

Both *Acinetobacter* strains described here may utilize the enzymatic pathway to biodegrade OTA that has been recognized in other microorganisms [22], degrading OTA by means of the hydrolysis of the OTA amide group and the consequent release of OTα, that is considered non-toxic [54,55]. Different peptidases are known to catalyze the above hydrolysis, such as the carboxypeptidase A enzyme isolated from the bovine pancreas [56], a commercially available lipase-like enzyme [57], an OTA-degrading protein enzyme from *Aspergillus niger* [58], the carboxypeptidase Y isolated from *Saccharomyces cerevisiae* [22], and a carboxypeptidase-like from *Trichosporon mycotoxinivorans* [29].

On the above basis, the biological degradation of OTA is a very feasible approach for the decontamination of foodstuffs, since the use of chemical or physical tools is likely to also eliminate, along with the mycotoxin, nutrients and organoleptic-relevant compounds. The possibility to reduce OTA levels by the here described *Acinetobacter* strains isolated from agricultural soils is of great interest for the control of OTA contaminations. In fact, these strains will be a source of efficient enzymes for OTA biodegradation in other food or feeds. Accordingly, further studies are in progress to determine which enzymes take part in the detoxification process by *Acinetobacter* sp. strain neg1, and to establish the absence of byproducts with any residual toxicity. Moreover, experiments are now underway to characterize and to clone gene(s) responsible for the detoxification reaction, in order to develop a novel enzymatic detoxification system.

## 4. Experimental Section

### 4.1. Soil and Branch Sampling from Vineyards

Soil and branch samples were collected from five vineyards of Negroamaro and Primitivo grape cultivars, in the most significant production areas for these varieties in Salento (Apulia, Southern Italy). In particular, three vines planted with the variety Negroamaro were trained in a bower system in a head system, or in spur-pruned cordon, and two vineyards planted with the variety Primitivo were trained in a bower system or in an espalier system (Table 2). All of the sampled vineyards were conducted by the methods of organic agriculture and reported to be contaminated by *A. carbonarius* and by high levels of OTA in previous years [59]. In each vineyard, during the “dormant” period, a random sampling of soil and branches was performed from each source. The samples were placed in sterile plastic bags, cooled, and analyzed within 4 h after sampling.

**Table 2:** Sampling from different ecological niches of the “system vineyard”.

Sample ID	Origin	Vineyard n.	Cultivar	Site	Training System
1 NG	soil	1	Negroamaro	Nardò (LE)	Bower system
1 NGT	branches	1	Negroamaro	Nardò (LE)	Bower system
2 PR	soil	2	Primitivo	Nardò (LE)	Bower system
2 PRT	branches	2	Primitivo	Nardò (LE)	Bower system
3 PR	soil	3	Primitivo	Manduria (TA)	Espalier
3 PRT	branches	3	Primitivo	Manduria (TA)	Espalier
4 NG	soil	4	Negroamaro	Cellino S. Marco (BR)	Head system
4 NGT	branches	4	Negroamaro	Cellino S. Marco (BR)	Head system
5 NG	soil	5	Negroamaro	Brindisi	Spur-pruned cordon
5 NGT	branches	5	Negroamaro	Brindisi	Spur-pruned cordon

### 4.2. Evaluation of the OTA-Degrading Ability of Soil and Branch Samples and Isolation of Microorganisms

Five grams of each sample (Table 2) were suspended in 50 mL of sterile distilled water. The suspension was incubated at room temperature with shaking (180 rpm) for 20 min. An aliquot (0.5 mL) of the suspension was added to 4.5 mL of three liquid substrates.

The three different liquid substrates used to support the growth of the microorganisms were: minimal medium (MM = K_2_HPO_4_ (2.5 g/L), KH_2_PO_4_ (2.5 g/L), (NH_4_)_2_HPO_4_ (1.0 g/L), MgSO_4_·7H_2_O (0.2 g/L), FeSO_4_ (0.01 g/L), MnSO_4_·7H_2_O (0.007 g/L)) with Bacto Peptone (0.5%, *w*/*v*) (MMP), minimal medium with sucrose (1.0%, *w*/*v*) (MMS), and potato dextrose broth (PDB) (Oxoid, Basingstoke UK) [35]. OTA (Sigma-Aldrich, St Louis, Missouri, USA) was added to MMP, MMS, and PDB media at a final concentration of 10 µg/mL. When the mycotoxin was used as the sole carbon source, it was added to MM medium at a final concentration of 100 µg/mL. Cultures were incubated at 24 °C with continuous shaking (180 rpm) and aliquots (0.5 mL) from each culture were collected after 0, 72, and 144 h. Cells were harvested by centrifugation (10,000× *g* for 10 min at 4 °C), the supernatants were filter sterilized (0.2 µm, Millipore, Billerica, MA, USA) and used for chemical analyses [60]. Each sample was tested in triplicate.

In order to assess the type of the OTA-degrading microbial populations, soil samples with OTA-degrading ability were treated as follows: an aliquot (0.5 mL) of the soil suspension, prepared as above described, was added to 4.5 mL of PDB and MMP medium, respectively, added with 50 µg/mL of chloramphenicol (for bacteria) or nystatine (for fungi), in the presence of 10 µg/mL of OTA. The amount of OTA biodegradation was determined after five days of incubation at 24 °C with continuous shaking (180 rpm). Moreover, to isolate OTA-degrading microorganisms, after three days of incubation, the cultures showing OTA-degrading ability were serially diluted and plated in triplicate on MMP agar. Plates were incubated for 24 h at 24 °C. From each samples, approximately 100 colonies were singly picked up from the agar plates and checked for purity prior to storage at −80 °C.

### 4.3. rep-PCR

In order to perform a molecular characterization of isolates, bacterial DNA was extracted from overnight cultures grown in Brain Heart Infusion (BHI) broth (Becton, Dickinson and Company, Franklin Lakes, NJ, USA) at 26 °C, using the Wizard Genomic DNA Purification Kit (Promega, Madison, WI, USA) and analyzed by repetitive element sequence-based PCR (rep-PCR) [61,62]. In the first phase of the study, in order to identify primers able to provide the best results in discriminating the strains, different primers were used to amplify the DNA of 12 isolates.

In particular, the primer pair REP-1R-Dt/REP-2R-Dt and the primer (GTG)_5_ were evaluated (Table 3). The PCR reactions and the PCR product analyses were performed as previously described [63]. The primer pair REP-1R-Dt/REP-2R-Dt discriminated better than the primer (GTG)_5_ the different strains, so it was chosen for the study of all the isolates.

**Table 3:** Nucleotide sequences of the different primers used in this study for rep-PCR and for amplification and sequencing of 16S rRNA gene.

Primer	Primer Sequence	Reference
REP-1R-Dt	5′-IIINCGNCGNCATCNGGC-3′	[63]
REP-2R-Dt	5′- NCGNCTTATCNGGCCTAC-3′	[63]
(GTG)_5_	5′-GTGGTGGTGGTGGTG-3′	[64]
P0	5′-GAGAGTTTGATCCTGGCTCAG-3′	[65]
P6	5′-CTACGGCTACCTTGTTACGA-3′	[65]
27f-YM	5′-AGAGTTTGATYMTGGCTCAG-3′	[66]
1492r	5′-TACCTTGTTACGACTT-3′	[66]
ENT-16S-for	5′-CAGCCACACTGGAACTGAGA-3′	This study
ENT-16S-rev	5′-GACAGCCATGCAGCACCT-3′	This study
ENT-16S-revII	5′-TTATGAGGTCCGCTTGCTCT-3′	This study
PSE-16S-for	5′-GGTCTTCGGATTGTAAAGCAC-3′	This study
PSE-16S-rev	5′-GACGACAGCCATGCAGC-3′	This study

### 4.4. Identification of Bacterial Strains

Isolates representative of each rep-PCR profile were identified by 16S rRNA gene sequencing [67] The universal primer pair P0/P6 or when appropriate the primer pair 27f-YM/1492r (Table 3) were used for 16S rRNA gene amplification and their locations at the 5′ and 3′ ends of the 16S rRNA gene allow to amplify almost all the gene. Each 50 μL reaction mixture contained 5 μL of 10× AccuPrime™ Pfx Reaction Mix, 1.25 U of AccuPrime™ Pfx DNA polymerase (Thermo Fisher Scientific, Waltham, MA, USA), 0.3 μM of each primer and 1 μL of genomic DNA. PCR amplifications were performed in a GeneAmp PCR system 9700. The reaction mixtures were first incubated for 2 min at 95 °C, and then cycled for 30 cycles according to the following temperature profiles: 30 s at 95 °C, 30 s at an annealing temperature of 60 °C for the first five cycles, 55 °C for the next five cycles and 50 °C for P0/P6 or 48 °C for 27F-YM/1492r for the last 25 cycles, then 3 min at 68 °C, followed by a final extension for 10 min at 68 °C. Sequencing of the 16S rRNA gene PCR products were performed in both forward and reverse directions by using the universal primers P0, P6, 27f-YM, 1492r and internal primers (Table 3). PCR products were sequenced by using the BigDye™ Terminator cycle sequencing kit on an ABI Prism 3730xl DNA Analyzer (Thermo Fisher Scientific, Waltham, MA, USA). The Blast N program, available through the National Center for Biotechnology Information [68] was used to compare the sequenced PCR products with sequences of type strains in database (“*Sequences from type material*” *option*). Bacterial strains were assigned to the species on the basis of the highest scores of alignment and percentage of identity (>99%) between their 16S rRNA gene sequences and those of type strains in database.

### 4.5. Biodegradation of OTA by Bacterial Isolates

Bacterial isolates were analyzed to evaluate their OTA-degrading ability. Isolates were cultured (2% *v/v*) in BHI broth and incubated at 24 °C with shaking (120 rpm). After overnight incubation, 5 mL of MMP containing 1 µg/mL of OTA were inoculated with subcultures (OD_600nm_: 1.0) at 2% (*v/v*) in triplicate. Controls were prepared by dissolving 1 µg/mL of OTA in MMP liquid in triplicate (100% OTA). Both inoculation and control tubes were incubated at 22, 24, and 28 °C with shaking. After one, three, and six days, 0.5 mL aliquots were collected, cells were harvested by centrifugation (10,000× *g* for 10 min at 4 °C), the supernatants were filter sterilized (0.2 µm, Millipore) and used for chemical analyses.

### 4.6. Preparation of Standards

OTA stock solution was prepared by dissolving the solid commercial toxin (Sigma-Aldrich, St. Louis, MO, USA) in methanol (1 mg/mL). Appropriate aliquots of the stock solution were brought to dryness and reconstituted with acetonitrile-water-acetic acid (99:99:2 *v*/*v*/*v*) to obtain standard solutions of OTA over the range 0.05–0.10 µg/mL. The standard of OTα was purchased from Biopure (Romer Labs Diagnostic GmbH, Austria) at a concentration of 10 µg/mL in acetonitrile. Aliquots of the stock solution were brought to dryness and reconstituted with acetonitrile-water-acetic acid (99:99:2, *v*/*v*/*v*) to obtain standard solutions of OTα from 0.01 to 0.10 µg/mL.

### 4.7. Chemical Analyses

*HPLC-FLD.* Decimal dilutions of the supernatants were made with acetonitrile-water-glacial acetic acid (99:99:2, *v*/*v*/*v*; Baker, Italy). Samples were filtered using RC 0.2 µm micro spin filter tubes (Phenomenex, Torrance, CA, USA). Fifty µL were injected into the HPLC apparatus (Technology series 1100, Agilent, Santa Clara, CA, USA) with a full loop injection system. Direct injection of liquid culture was possible since no interfering peaks eluted at retention times of OTα and OTA.

Measurement of toxins was carried out as described by Gallo *et al.* [69], with minor modifications. OTα and OTA were measured by comparing peak areas with calibration curves; the retention times were 7.75 and 10.36 min, respectively.

The detection limit was 0.1 ng/mL for OTA and OTα (based on a signal-noise ratio 3:1). *HPLC-HRMS.* Structural confirmation of OTα and identification of others possible degradation metabolites were carried out by LC-HRMS. Twenty microliters of supernatants were also analyzed by LC-HRMS according to the procedure reported by Gallo *et al.* [69].
